# P19 IV antimicrobial duration in practice

**DOI:** 10.1093/jacamr/dlac004.018

**Published:** 2022-02-16

**Authors:** Abisoye O. Akintimehin, Paul Wade, Aisling F. Brown

**Affiliations:** Department of Infectious Diseases, Guy’s and St Thomas’ NHS Foundation Trust, London, UK; Department of Infectious Diseases, Guy’s and St Thomas’ NHS Foundation Trust, London, UK; Department of Pharmacy, Guy’s and St Thomas’ NHS Foundation Trust, London, UK; Department of Infectious Diseases, Guy’s and St Thomas’ NHS Foundation Trust, London, UK

## Abstract

**Background:**

Tackling antimicrobial resistance by reducing unnecessary or inappropriate prescribing is a key priority.

**Objectives:**

To review current clinical practice at Guy's and St Thomas’ Hospital around duration of IV antimicrobial therapy framed with Start Smart Then Focus national guidelines.

**Methods:**

Newly started inpatient prescriptions for IV antimicrobials over three consecutive Mondays were reviewed. Prescriptions not for treatment of acute infections were excluded, as were patients admitted to critical care, obstetrics/gynaecology and paediatric wards. Each patient's record was reviewed at Day 0, 3 and 5. Data were analysed using IBM® SPSS Statistics version 27.

**Results:**

In total, 80 patients with 89 antibiotic prescriptions (71 = β-lactam, 5 = aminoglycoside, 2 = glycopeptide, 1 = ciprofloxacin, 9 = metronidazole, 1 = linezolid) were included. Median age was 66 (IQR 53–75). Documented indications were for suspected or proven infection of urinary tract (15%), respiratory tract (25%), skin/soft tissue (14%), sepsis (1.25%), intra-abdominal (15%), bacteraemia (7.5%), bone and joint infections (2.5%) or other (20%). Median initial NEWS2 on Day 0 was 3 (IQR 1–4), WCC 11.45 (IQR 8.27–14.15) and CRP 121 (IQR 47–208). Median length of stay was 7.5 days. Median duration of IV antimicrobial was 4 (IQR 2–6) days. By D3, 3 patients had died and 16 had been discharged. Of 61 remaining inpatients on D3, 4 had their antimicrobials switched, 14 had been switched to PO antimicrobials, 43 were continued on IV antibiotics. By D5, 17 further patients had been discharged, 44 remained inpatients. Twenty-two had stopped antimicrobials with 22 continuing IV therapy. A number of patients could have been switched from IV to oral therapy by current guidance but were not (D3 x = 11, D5 n = 8). Higher median D0 CRP was seen in those who continued on IV therapy on D3 (139 versus 79 mg/dL) as was higher D3 WCC (13.9 versus 11.9 × 10^9^/L). There was no significant relationship between a NEWS2 score ≥3 and IV to PO switch at D3 or D5. Thirty-two patients had input from the Infection team within 7 days of first prescription. Fifteen patients (21%) received IV antibiotics for more than 7 days, and 14/15 had Infection team input. Three patients were discharged with OPAT.

**Conclusions:**

Median duration of IV therapy for suspected/proven infections in our centre is less than 5 days and only 28% of patients were still receiving IV antibiotics by D5. A number of missed opportunities for earlier switch to oral therapy were identified. The potential for earlier safe discontinuation of IV therapy using patient-specific measures should be explored. A minority of patients received IV therapy for >7 days, and this was almost universally associated with specialist Infection involvement in complex infections with a view to optimizing patient management and antimicrobial stewardship.

